# Can public sector community health workers deliver a nurturing care intervention in South Africa? The *Amagugu Asakhula* feasibility study

**DOI:** 10.1186/s40814-021-00802-6

**Published:** 2021-02-27

**Authors:** Sonja Klingberg, Esther M. F. van Sluijs, Stephanie T. Jong, Catherine E. Draper

**Affiliations:** 1 MRC Epidemiology Unit, UKCRC Centre for Diet and Activity Research (CEDAR), University of Cambridge, Cambridge, UK; 2 SAMRC/Wits Developmental Pathways for Health Research Unit (DPHRU), School of Clinical Medicine, Faculty of Health Sciences, University of the Witwatersrand, Johannesburg, South Africa; 3 Faculty of Medicine and Health Sciences, School of Health Sciences, University of East Anglia, Norwich, UK; 4 Division of Exercise Science and Sports Medicine, Department of Human Biology, Faculty of Health Sciences, University of Cape Town, Cape Town, South Africa

**Keywords:** Nurturing care, Community health workers, South Africa, Qualitative research, Process evaluation, Feasibility study

## Abstract

**Background:**

Nurturing care interventions have the potential to promote health and development in early childhood. *Amagugu Asakhula* was designed to promote developmentally important dietary and movement behaviours among children of preschool age (3–5 years) in South Africa. An initial formative study in Cape Town found the intervention to be feasible and acceptable when delivered by community health workers (CHWs) linked to a community-based organisation. This study evaluated the delivery of the *Amagugu Asakhula* intervention by CHWs linked to a public sector primary health care facility in Soweto, as this mode of delivery could have more potential for sustainability and scalability.

**Methods:**

A qualitative design was utilised to assess feasibility, acceptability, adoption, appropriateness, implementation, fidelity and context. CHWs (*n* = 14) delivered the intervention to caregivers (*n* = 23) of preschool-age children in Soweto over 6 weeks. Following the completion of the intervention, focus group discussions were held with CHWs and caregivers. Further data were obtained through observations, study records and key informant interviews (*n* = 5). Data were analysed using deductive thematic analysis guided by a process evaluation framework.

**Results:**

The delivery of the *Amagugu Asakhula* intervention through CHWs linked to a primary health care facility in Soweto was not found to be feasible due to contextual challenges such as late payment of salaries influencing CHW performance and willingness to deliver the intervention. CHWs expressed dissatisfaction with their general working conditions and were thus reluctant to take on new tasks. Despite barriers to successful delivery, the intervention was well received by both CHWs and caregivers and was considered a good fit with the CHWs’ scope of work.

**Conclusions:**

Based on these findings, delivery of the *Amagugu Asakhula* intervention is not recommended through public sector CHWs in South Africa. This feasibility study informs the optimisation of implementation and supports further testing of the intervention’s effectiveness when delivered by CHWs linked to community-based organisations. The present study further demonstrates how implementation challenges can be identified through qualitative feasibility studies and subsequently addressed prior to large-scale trials, avoiding the wasting of research and resources.

**Supplementary Information:**

The online version contains supplementary material available at 10.1186/s40814-021-00802-6.

## Key messages regarding feasibility

- Integrating nurturing care into existing systems has been recommended in low- and middle-income countries with scarce resources for scaling up interventions, but there were uncertainties regarding the feasibility of public sector community health workers delivering such interventions in South Africa.
- Our qualitative findings illustrate how contextual challenges may hinder such integration, as intervention delivery by public sector community health workers was not found to be feasible.
- Delivery by community health workers linked to community-based organisations instead of primary health care facilities is recommended for future trials of the *Amagugu Asakhula* intervention.

## Background

Nurturing care has become increasingly recognised as a key framework for early childhood development [1]. Defined as “a stable environment that is sensitive to children’s health and nutritional needs, with protection from threats, opportunities for early learning, and interactions that are responsive, emotionally supportive, and developmentally stimulating” [1] (p. 91), nurturing care largely aims to ensure that children grow and develop to reach their full potential. Nurturing care interventions in early childhood are a promising avenue for addressing a multitude of child health and developmental challenges, such as different forms of malnutrition and developmental delay, in low- and middle-income countries (LMICs) [2, 3].

The importance of promoting early childhood development is well established and widely recognised, but the implementation and scale-up of interventions that can achieve such outcomes in LMIC settings need to be enhanced [4, 5]. In particular, it is important to understand dimensions such as delivery, feasibility, context, and integration of interventions into existing systems [4, 6]. These dimensions can be examined through process evaluations and formative research, which are recommended components of intervention development and testing [7, 8]. Formative studies, such as pilot and feasibility studies, provide research insights at a small scale to help to determine whether an intervention is appropriate for further testing [9], enable optimisation of interventions prior to larger evaluations or wider implementation, and support decisions regarding definitive effectiveness trials [10–12]. The embedment of process evaluations and formative research, particularly in implementation research in LMIC settings, has been called for in order to maximise learning about interventions and avoid wasting scarce resources [5, 13, 14].

Following the success of a South African paediatric HIV disclosure intervention called *Amagugu* [15–17], the intervention was adapted into an early childhood health and development intervention called *Amagugu Asakhula* (“treasures that are still growing”). While certain key elements of the original *Amagugu* intervention were retained (caregiver support, strengthening the caregiver/child relationship, counselling approach, and home-based delivery), the original HIV messages were replaced with messages focussing on children’s cognitive development, physical activity, screen time, diet, and sleep. This adapted intervention aligns with the nurturing care framework [1], and its theory of change is described in detail elsewhere [3]. *Amagugu Asakhula* is delivered by Community Health Workers (CHWs) to individual caregivers of preschool-age children through weekly sessions over a six-week period. It intends to promote nurturing interactions and developmentally important health behaviours.

An initial pilot study of the *Amagugu Asakhula* intervention in a low-income urban setting in Cape Town, South Africa, found it to be both feasible and acceptable for caregivers of preschool children in a low-income urban setting, when delivered by CHWs linked to a community-based organisation [3]. Based on this initial formative work, the *Amagugu Asakhula* intervention was deemed appropriate for further testing. The present study aimed to evaluate an alternative delivery mode through CHWs linked to a public primary health care (PHC) facility in Soweto, a predominantly low-income, urban setting in South Africa. Through making use of existing public sector structures, this delivery mode could have considerable potential for future implementation, including opportunities for scale-up, cost-effectiveness and sustainability of the intervention, if *Amagugu Asakhula* is found to be effective.

Community-based PHC settings in LMICs are a potential entry point for nurturing care interventions, and the integration of new interventions with such existing platforms is recommended [1]. Public PHC in South Africa is organised around clinics and community health centres that are free at the point of use [18]. Through CHWs, PHC facilities can also carry out health promotion activities and community outreach and link with other public providers such as social services. Public sector CHWs are part of a ward-based outreach team (WBOT) system, which means that they are deployed in their local communities and attached to a public PHC facility [19]. CHWs are each responsible for a specified number of households in their community, and their tasks include, for instance, promoting antenatal care attendance, the immunisation of children, and treatment adherence in the case of chronic illnesses. CHWs are managed by team leaders who are trained nurses [20, 21] and receive formal training before being deployed in the communities. While some formalised processes are in place, there are notable gaps between policy and implementation in terms of the WBOT system and PHC more generally [19, 22]. For example, linkages and referrals between different institutions and services are suboptimal in many cases, and there has been an insufficient focus on prevention and health promotion [22].

The primary aim of the current study was to evaluate the feasibility and acceptability of the delivery of *Amagugu Asakhula* by CHWs linked to a public PHC facility in Soweto, a predominantly low-income, urban setting in South Africa. A further aim was to generate context-specific insights about implementation to support the optimisation of the intervention and its delivery in a new setting.

## Methods

### Study design and setting

**Fig. 1:**
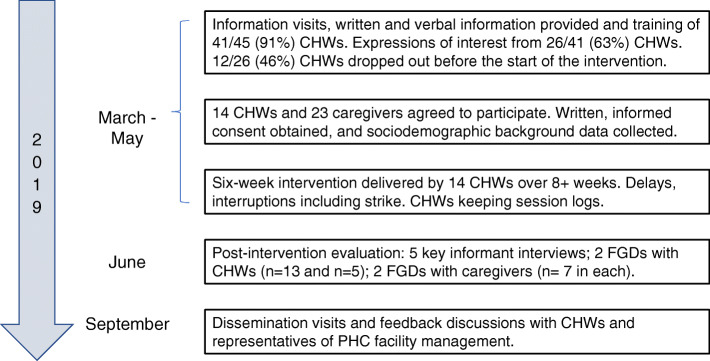
Flowchart of the *Amagugu Asakhula* feasibility study

This feasibility study examined feasibility and acceptability, as well as adoption, appropriateness, implementation, fidelity, and context [12, 15, 16]. Data collection methods included semi-structured key informant interviews, focus group discussions (FGDs) with CHWs and intervention participants, qualitative observations, sociodemographic background questionnaires, and tracking of recruitment, training, and implementation records. This study is reported in accordance with the TIDIeR and SRQR reporting guidelines [23, 24], as well as the consolidated advice for reporting early childhood development implementation research (CARE) guidelines [25]. The study process is illustrated in Fig. 1.

A specific PHC facility in Soweto was selected as the site for this study due to existing contacts with the facility and the site’s openness to engage with research. Due to the sensitive nature of some findings and to preserve confidentiality, the facility in question and its location in Soweto will not be identified. Soweto is a densely populated urban township in Gauteng Province, South Africa. Due to the legacy of colonial and apartheid urban planning and policies of racial segregation, Soweto’s population is predominantly Black African. Some socioeconomic diversity exists, most notably between very deprived areas considered informal settlements, and more formal middle-class neighbourhoods. The study setting was a predominantly low-income area with formal housing, characterised by permanent structures such as brick and cement houses, as opposed to more temporary structures such as shacks made of corrugated iron.

While the design of this feasibility study in Soweto is similar to that of the original pilot study in Cape Town [3], there are key differences in intervention delivery that warrant investigation. In Soweto CHWs were linked to a public PHC facility, while in Cape Town CHWs were linked to a community-based organisation. In Soweto, CHWs delivered the intervention individually, while in Cape Town CHWs worked in pairs due to different safety concerns [3]. In addition, CHWs in Soweto only delivered the intervention to one or two caregivers, while each pair of CHWs in Cape Town was required to deliver the intervention to ten caregivers. CHWs in Cape Town had a lower existing workload than CHWs in Soweto linked to a public PHC facility.

Ethical approval was obtained from the University of the Witwatersrand’s Medical Human Research Ethics Committee (reference number M181063). All participating CHWs and caregivers gave informed written consent to participation and to being audio-recorded if relevant. Permission to carry out the study through a public PHC facility was also obtained from the Johannesburg Health District Research Committee (DRC Ref. 2019-01-013). After formal permission to conduct research at the facility had been obtained, SK provided information about the intervention and study to all CHWs, their managers and team leaders via information sheets. All CHWs and team leaders linked to the facility were invited to take part in the *Amagugu Asakhula* training sessions that prepared CHWs to deliver the intervention to families with preschool-age children.

### Training and recruitment

CHWs attended two 4-h training sessions delivered by SK who had attended the training of CHWs in Cape Town and been trained by one of the intervention developers to deliver CHW training. The training was delivered in a seminar format with considerable practical examples and ample time for questions and discussions. The training sessions introduced the research aspects of the study, covered background information about child nutrition, health, and development, as well as the approach, content, and materials of the *Amagugu Asakhula* intervention. Topics were also detailed in an instruction manual that was distributed to each CHW. Considerable focus was given to health-related behaviours according to South African guidelines on movement and dietary behaviours relevant for this age group [26–29] and how these relate to child nutrition, obesity prevention, and children learning and developing well. Expectations of participating CHWs, information, and materials were described in detail, both verbally and in the manual, along with the activities for CHWs to deliver to caregivers in each of the six sessions. Some individual tailoring was encouraged to communicate with caregivers in a relevant and respectful way. For example, when covering healthy dietary behaviours, CHWs were asked to align the discussions with each family’s perceived circumstances and level of food security. Refreshments, including lunch, were provided to CHWs and team leaders attending the training sessions. CHWs who attended the training received certificates of attendance.

CHWs who attended training were asked to register their interest and intention to deliver the intervention. We employed a pragmatic approach to enable the budgeting of intervention materials for packs that would be purchased and distributed to CHWs. At the end of the second training session, information sheets, consent forms, and sociodemographic questionnaires were provided to CHWs in order for them to recruit caregivers of preschool-age children into the study. CHWs had 1 week to make contact with and recruit caregivers prior to delivering the intervention. All participating caregivers, and all CHWs who completed the intervention, were given ZAR200 (~US$ 11) supermarket vouchers as appreciation for their time and input to the research.

### Study procedures and data collection

The entire study was carried out between March and September 2019. The process is described in Fig. 1. To complete the intervention, CHWs were required to attend at least one training session and deliver six sessions over 6 weeks to the caregivers they recruited. As this was a pragmatic evaluation, participating CHWs were responsible for recruiting caregivers, obtaining informed written consent for the caregivers’ participation, and background sociodemographic characteristics of participating caregivers (see Additional files). They were also asked to keep records of home visits and write brief notes after each intervention session using printed forms with specific questions (see Additional files).

Three weeks after the 6-week intervention had been completed, SK conducted five key informant interviews with individuals who had insights into the organisational structures and systemic dimensions of the WBOTs. The informants held positions of responsibility within the daily operations of the PHC facility. To preserve confidentiality of the interviews and respect the anonymity of the five individuals, these participants are not described in detail.

In the same time period following the intervention, four FGDs were conducted: two with participating caregivers (*n* = 7 in each group), one FGD with CHWs who delivered the intervention (*n* = 13), and one FGD with CHWs who dropped out of delivering the intervention (*n* = 5). The FGDs were moderated by SK, with note-taking assistance from graduate students. All but one CHW delivering the intervention took part in the FGD. CHWs preferred to be in the same FGD, rather than divided into smaller groups. Refusals to participate in FGDs were due to other commitments (CHW, *n* = 1), not wanting to be audio-recorded (caregivers, *n* = 2), or not specified (caregivers, *n* = 7).

All interviews and FGDs were conducted in English by SK (see Additional files for instruments used) and audio-recorded (Philips DVT4010 VoiceTracer). They were transcribed verbatim either by SK (*n* = 2 FGDs with CHWs) or by a professional transcriber (*n* = 2 FGDs with caregivers and *n* = 5 interviews). All professionally generated transcripts were checked by SK against original recordings. Field notes of observations were kept by SK during all visits to the PHC facility. Records were kept of training and meeting attendance, recruitment and retention of CHWs and caregivers, and all correspondence between SK and participating individuals.

In September 2019, study findings were shared by SK and CED with participating CHWs and other stakeholders. Feedback regarding the study was discussed over 2 days.

### Evaluation framework and data analysis

Data analysis software MAXQDA (Release 12.2.0) was used to facilitate qualitative data analysis and management. Based on existing literature [7, 30], Table 1 outlines the applied process evaluation framework developed for evaluating the delivery the *Amagugu Asakhula* intervention.

**Table 1:** Process evaluation framework

Concept	Questions addressed	Data sources
Adoption	To what extent were the training (CHWs) and intervention (CHWs and caregivers) adopted by targeted groups?	Recruitment and retention records, observations, separate FGDs with CHWs, and caregivers after the intervention
Acceptability	To what extent was the intervention delivery agreeable and satisfying, and perceived as acceptable by caregivers and CHWs?	Separate FGDs with CHWs and caregivers after the intervention
Appropriateness	How well does the intervention correspond with caregivers’ situations and needs? Are CHWs well suited for delivering this intervention?	Separate FGDs with CHWs and caregivers after intervention, observations during training and intervention, feedback from CHWs about training, and key informant interviews
Implementation and fidelity	To what extent was the intervention implemented as intended in the design, and consistently with the underlying theory and philosophy?	Observations, key informant interviews, separate FGDs with CHWs, and caregivers after intervention
Feasibility and context	How successfully can this intervention be carried out in this setting? How successfully can the necessary evaluation components of this intervention be carried out in this setting? Are there contextual barriers or facilitators related to this intervention and/or its evaluation? To what extent are they modifiable, and do they necessitate further tailoring of the intervention for a full trial? Were there any factors external to the intervention that seemed to influence implementation?	Recruitment and retention records, observations, key informant interviews, separate FGDs with CHWs, and caregivers after intervention

Analysis followed the methods developed by Braun and Clarke for thematic analysis, but the analytic process comprised what can be called ‘codebook’ thematic analysis rather than the reflexive approach to thematic analysis [31–33]. Themes were developed a priori using a deductive (theoretical) approach, based on the process evaluation framework. Accordingly, the pre-developed themes were used as a starting point for the coding of both interview and FGD transcripts. Themes were divided into codes such as ‘in support of feasibility’ and ‘against feasibility’, and the proximity of the different participant types to the intervention informed the weight that was given to their views in forming conclusions. Coding was conducted by SK, and the results of the thematic analysis were discussed and refined by the full team of authors, utilising a ‘critical friends’ approach for enhancing rigour in the analysis [34]. Field observations were used to support interpretation and analysis.

## Results

Table 2 provides an overview of the results depicted as illustrative quotations and organised according to the process evaluation framework. The findings describe how different aspects of the *Amagugu Asakhula* intervention and its delivery were received by both participating caregivers and the CHWs delivering it. Most importantly, it details barriers and facilitators to the specific intervention delivery approach, namely through public sector CHWs.

**Table 2:** Overview of results of the *Amagugu Asakhula* feasibility study in Soweto

Concept	Conclusion	Illustrative quotations
Adoption	Mixed results.	“I thought maybe it’s like just too much work and everything.” (CHW who dropped out of the intervention delivery, FGD) “Everybody at first is sceptical (others murmuring in agreement) about the programme, and then when you get to learn about what the programme offers then you start enjoying it.” (Caregiver, FGD) “Even sister [name of CHW] was good to me, because she came home and she introduced this thing to me and then she explained everything to me. Even though I was like with, like her, because I was sceptical like *hayi* [no]. (laughing, others laughing too) I don't have time to sit down and do this thing. But she, she made me understand that it is not for me, it is for me and my child (someone else says "yes" in the background).” (Caregiver, FGD) “When they came and they say like it's like a crèche [preschool], *nè* [right], you are teaching your child and all that and in your mind you are thinking ‘Ah I don't have time for that like, how do I teach a child this and that?’ You understand. So, I mean at first *nè*…I was not really interested, I was just doing [name of CHW] a favour. To be honest.” (Caregiver, FGD)
Acceptability	Acceptable to both CHWs and caregivers.	Moderator: “So what about the fact that the community health workers were coming to your homes with this programme…?” Caregiver 1: “I prefer home visits. Because it's drawing attention to the other kids as well. I can teach my neighbour’s child as well.” (…) Moderator: “Did any of you find it somehow uncomfortable that these were home visits? Or any other comments or feedback?” Caregiver 2: “Not really, because they do visit.” (…) Caregiver 3: “[The CHWs] should come. It’s something we are used to. Because they do the home visits, so it was very nice doing something different…” (laughter) Caregiver 4: “Like ‘How are you feeling today, did you take your medicine?’ This [intervention] was like WOW. Something else. It was nice.” (FGD) Interviewer: “What about your impressions of how acceptable it was for the community health workers to do this type of, you know, longer home visits and several sessions and actually really going through almost teaching things…? How acceptable is it to do that kind of tasks compared to what the normal work is?” Key informant: “Uh huh (laughs) well it's, to be truthful, it was easy. Why I’m saying it was easy, because…our scope of practice is to educate the families, to educate the individuals, is to educate, educate… So it was easy for us because we already engaged with those houses. We already knew where to go to assist, and we already knew people, who to talk to, who to educate to, who’ve got children.” (Key informant interview)
Appropriateness	Appropriate for caregivers. The CHWs are also suited to deliver the intervention based on their skills and the scope of work.	“Then after [the CHW] told me that ‘This thing, it’s a project with kids, so I don’t know how you’re going to feel about it.’ Then I said ‘Of course I will try and force my children to have the better education and I want to see my children being happy.’” (Caregiver, FGD) “If it is done by community health workers I think it’s good because they are our foot soldiers to the community. That’s why they are sent… I think they are the good people for the [*Amagugu Asakhula*] programme.” (Key informant interview)
Implementation and fidelity	Mixed results. Some specific descriptions by caregivers indicated high fidelity of intervention activities and content, but CHWs had also made changes to intervention.	Caregiver 1: “Then I realised that raising a baby is like planting a seed [referring to an intervention activity involving the planting of seeds].” Caregiver 2: *“*Yes*.”* Caregiver 1: “You have to give it all your attention, take care of it and love it, so that is what I learned.” (FGD) “Here, all of us we were doing the training with the caregivers differently... You would have expected us to follow the manual exactly the things, ‘Tell the caregivers what will affect the brain development of a child…’ like follow exactly the manual. Yet we did those things in our own understanding and in our own ways where it helped us.” (Participating CHW, FGD)
Feasibility and context	Not feasible to be delivered by CHWs linked to a PHC facility. Only partially successful implementation. Challenges suggest evaluation components will be difficult to carry out through CHWs. Considerable organisational and structural barriers to integrating the intervention to the work of CHWs.	“Community health workers try to find every excuse not to do things and can walk around aimlessly just clocking up visits. That’s what’s happening.” (Key informant interview) “But the strike…it strains a lot of people, meaning most of the people didn’t want to go work anymore. Didn’t want to work, because they were thinking ‘Ah, the government is failing us again.’ Ah, you know. So it’s yeah, it kills our jobs, like yeah, yeah it affected our job like so badly, so badly.” (Key informant interview) “They [CHWs] can’t do [work] with an empty stomach... They need to get paid so that they have that energy so that they can do whatever.” (CHW who dropped out, FGD) “Yes, people will be like ‘Okay, will we get extra money for it?’ (sighs) That’s not motivation, that’s being selfish.” (Key informant interview)

### Adoption

In total, 91% (41 out of 45) of CHWs attended at least one of the two training sessions; 18 attended both sessions. Subsequently, twenty-six CHWs expressed interest at this initial stage, agreeing to recruit 1–2 caregivers to deliver the intervention to over the subsequent 6 weeks. However, only 14 CHWs actually delivered the intervention, and 23 caregivers participated in the study.

Initial scepticism towards the intervention was reported by both CHWs and caregivers, which impacted upon their desire to participate in the intervention. Among CHWs, initially, the intervention was described as “extra work” or “too much work”. Among caregivers, reasons for their initial scepticism included concerns about the required time commitment, perceptions that the intervention was “boring”, the expectation of having to teach children things, learning how to raise children despite already having experience of raising children, and fears about the intervention putting children at risk of strangers. CHWs were able to mitigate these reservations in two ways: (1) using the existing relationships and trust built between CHWs and caregivers to reassure them about the study (Table 2) and (2) by providing more information. Children’s interest in the intervention activities prompted caregivers to overcome their initial concerns, and they demonstrated more enthusiasm about *Amagugu Asakhula*.

It was also reported in both interviews and FGDs that there were financial barriers to adoption of the intervention. Key informants suggested that both CHWs and caregivers were likely to want incentives or compensation for participating in the intervention. Inability of the study to provide additional pay, on top of the challenges to CHWs receiving their regular salaries on time (unrelated to the study), reportedly played a considerable part in the lack of adoption from CHWs. CHWs who attended the training were informed that participation would not increase their salaries. As such, the reported delays in receiving their regular salaries may have been a more significant reason for the high number of CHWs who did not deliver the intervention after completing a training session (*n* = 12).

### Acceptability

Caregivers did not express any objections to the intervention being delivered by CHWs and were enthusiastic about the convenience of the sessions taking place in their homes. Many of the participating caregivers were unemployed and described having plenty of time for the home-based intervention. Some caregivers described instances where CHWs turned up unannounced, causing some inconvenience. However, these situations were usually resolved by agreeing on specific times for *Amagugu Asakhula* sessions.

While it was normal for CHWs to visit families in the community, one key informant expressed concerns about having CHWs visit specific homes on a frequent basis as a potential barrier to acceptability from the caregivers’ perspective due to the potential stigma attached to such visits. However, this concern about was not expressed by any caregivers, some of whom described telling neighbours about *Amagugu Asakhula*, and doing intervention activities with other children in their neighbourhoods. In turn, caregivers suggested incorporating group sessions or activities to the intervention itself. Caregivers expressed that participating in the FGD was a positive experience as it provided them the opportunity to hear about other families’ experiences.

### Appropriateness

Both CHWs and key informants reported that the *Amagugu Asakhula* intervention was a good fit with the regular scope of CHWs’ work, and that they were able to carry out home visits thanks to being known and trusted by community members. According to key informants and CHWs themselves, CHWs linked to a PHC facility should, at least in theory, be able to accommodate this type of intervention within their work both in terms of both its content and focus on preschool-age children, as well as the home-based mode of delivery.

Similarly, the intervention resonated with the caregivers’ situations as they were able to carry out intervention activities with their children. Caregivers described how the intervention aligned with their aspirations to ensure their children could learn well and be happy (Appropriateness and acceptability, Table 2). Caregivers who could not afford to send their children to preschool were particularly keen to support their children’s learning and development through the activities and nurturing interactions promoted through *Amagugu Asakhula*. Some caregivers reported being surprised by how much their young children had learned in a short amount of time, and this sense of achievement, combined with the children’s enjoyment, was central to the appeal of the intervention.

### Implementation and fidelity

The delivery of the 6-week intervention was interrupted after the first week due to CHW strikes taking place across South Africa. During the 2 weeks that strikes took place, no *Amagugu Asakhula* sessions were run. Many of the CHWs reported not working during this time. Due to the delays, it took at least 8 weeks for CHWs to complete the sessions with participating caregivers. Data collection for the post-intervention evaluation was carried out 3 weeks after intervention delivery was completed.

Study findings denote differences in the delivery of *Amagugu Asakhula* compared to the intended design of the intervention. Based on caregivers’ detailed descriptions of activities, and reflections that they shared with each other in FGDs, there were indications that many activities and topics had been covered by CHWs according to the manual. However, there was also some evidence that recruitment and delivery did not go according to the intervention’s intended procedures and criteria. For example, some caregivers who participated in FGDs did not have children in the targeted age range, despite the confirmation from sociodemographic questionnaires returned by CHWs. Moreover, during the FGDs, it emerged that some CHWs had not yet started the intervention with participants by the time they had been invited to post-intervention FGDs. Due to the discrepancies in session records, the CHWs’ accounts of sessions completed have been excluded as evidence of fidelity.

In addition to these indications of low fidelity, CHWs also reported in FGDs that they had made changes to the intervention. These changes included asking caregivers to study the intervention manual on their own, covering some sessions via WhatsApp messages or phone calls rather than in person, and skipping or combining some sessions. Some of these changes had been made in response to specific challenges, such as difficulties scheduling the weekly sessions. CHWs reported that the lack of close monitoring of the CHWs’ activity enabled them to do things in their preferred way, as opposed to how the intervention had been intended.

CHWs discussed how it was possible for them to personalise the content, and communicate intervention messages in a way that they found helpful, as opposed to exactly how they were described in the intervention manual (Table 2). However, despite it being covered in the training provided, CHWs described that it was unclear which aspects of the intervention were flexible (e.g. how the content is phrased) and which aspects were not (e.g. inclusion criteria, and the number, order, delivery method, and focus of sessions with participating families).

### Feasibility and context

As mentioned, both CHWs and key informants considered the intervention a good fit with the regular work and roles of CHWs linked to a public PHC facility in Soweto, at least in theory. Some key informants affirmed that the *Amagugu Asakhula* intervention could be scaled up and integrated into the WBOT system if found to be effective in the future. However, the CHWs’ reluctance to have new or additional work incorporated into their existing roles presented a considerable barrier to feasibly implementing the *Amagugu Asakhula* intervention. Similarly, key informants who openly acknowledged this barrier were sceptical of how implementation could be done in practice. Thus, there were some contradictions both within and between the key informants’ and CHWs’ accounts.

Two clashing narratives about the challenges were expressed by CHWs and key informants respectively. CHWs emphasised that they had too much to do, while key informants indicated that the CHWs were not doing enough in their role. CHW’s view of being overworked for their current salaries was closely linked to the strikes and labour union action taking place across the country at the time of the intervention. CHWs were unhappy with their precarious status; some reported that their contracts defined them as interns or trainees, rather than employees, and many were disgruntled with either their salaries, or not being paid on time. According to CHWs, introducing any additional or new work to their role would require an addition in salary, and strengthening their status as public sector health workers. The contrasting view denotes that CHWs are not monitored well enough. Due to this lack of accountability, as well as a perceived lack of motivation, commitment or work ethic, CHWs were thought of as doing less work than could reasonably be expected of them for their salary. As such, CHWs were perceived as being unwilling take on anything new.

While it is not possible to ascertain whether one of these views, a combination, or something else entirely, was the most accurate account of the situation, these organisational challenges and conflicts were described as considerable challenges to delivering *Amagugu Asakhula*, as well as the WBOT system more broadly. It was recommended by both CHWs and key informants that the intervention could or should be delivered through or together with other actors, such as preschools and community-based organisations.

## Discussion

This feasibility study has investigated the feasibility and acceptability of delivering the *Amagugu Asakhula* intervention through CHWs linked to a PHC facility in Soweto. The analysis indicates that the intervention and its delivery are both acceptable and, in theory, appropriate. However, due to contextual challenges, the delivery of the intervention is, in practice, not feasible via CHWs linked to a PHC facility. This is in line with other studies examining CHW systems in South Africa and other LMIC settings [20, 35–37], as CHWs have become a cadre of health workers to whom various new tasks are assigned, without necessarily much consultation or consideration of their capacity in terms of time, working conditions or skills.

The challenges of supervision and accountability around CHWs in South Africa have been previously recognised [5] and are not unique to South Africa. A study conducted in Pakistan similarly evaluated the integration of early childhood development interventions to an existing community health programme, and it highlights the importance of supportive CHW supervision, and the need for capacity building at the organisational level for improved implementation and scalability of the intervention [38]. The findings of the present feasibility study highlight the corresponding perspectives of the CHWs, who are hindered and demotivated by organisational issues, which understandably influence their performance. A systematic review of interventions to improve CHW performance in LMICs found potential for improvements through interventions such as tailored incentives, task reminders and an emphasis on career opportunities when recruiting CHWs [39]. However, the accounts of the Sowetan CHWs suggest that simply being paid on time and having more contractual stability might have a bearing on CHWs’ motivation and performance.

Some of the contextual challenges encountered in the present study provide an opportunity to optimise the intervention by, for example, modifying it to better accommodate delays and disruptions in the future. In particular, the strikes provided insight into the overall functioning of the WBOT system, and the PHC facility’s operations more generally, which paints a more complex and volatile picture. Views expressed by both key informants and CHWs in FGDs pointed to issues with supervision, accountability, resistance to change, and practical challenges of balancing work in the community with demands for being present in the facility. Prior research in South Africa has also identified the lack of supervision or monitoring of the CHW role as a challenge for the WBOT system [40].

The delivery challenges and hesitant adoption of the intervention by CHWs linked to a public PHC have implications for the implementation of *Amagugu Asakhula*. To ensure scalability and sustainability, existing public health system structures are recommended avenues for delivering interventions [1, 4]. Conversely, findings from the current study indicate that it may be more realistic to consider other options for delivering the *Amagugu Asakhula* intervention. Instead of public sector institutions, other health system actors, such as CHWs linked to community-based organisations, could deliver the intervention without being constrained by some of the challenges of the WBOTs. As the pilot study in Cape Town demonstrated, CHWs whose time is dedicated to the intervention, and compensated for, could deliver the intervention more successfully [3]. This route through community-based organisations is not without its own challenges, such as higher costs and possibly limited potential for scalability and sustainability compared to that of the public sector. Implementation of CHW programmes through community-based organisations has also been criticised for lacking accountability [20], and it ignores the need to the strengthen community health systems through the public sector [41, 42].

The recommended process of integrating nurturing care interventions into existing delivery systems [4], such as the WBOT system in South Africa, may become realistic to pursue further in the future if rigorous evaluations demonstrate the effectiveness of the *Amagugu Asakhula* intervention. This study not only provides valuable insights into how the contextual challenges influence implementation, but it also highlights the ways in which CHWs are embedded in the community, well accepted by caregivers, and preferred agents for delivering health interventions [43]. Supporting the wider efforts to strengthen community health systems while integrating new nurturing care elements into the WBOT scope of work may be a way to promote child health and development in the future, with more evidence and insights of the effectiveness of specific interventions.

While there were some evident challenges to adoption among both CHWs and caregivers, the reported phenomenon of eventually adopting and accepting the intervention after initial hesitation is an important finding for ensuring successful recruitment and adoption in future interventions. It further points to the importance of broader and earlier community and stakeholder engagement, comprehensive and clear information regarding the intervention, and the need for better understanding the determinants of participation in research or interventions in this setting [44]. Nevertheless, the positive views expressed by caregivers echo other research on nurturing care interventions in LMICs [4], as caregivers tend to value the focus on children’s well-being and learning in such interventions. Such findings are promising in terms of ensuring demand for the intervention if implemented more widely.

There are other practical lessons to be drawn from this study for future implementation and evaluation of *Amagugu Asakhula* or other interventions targeting early childhood health and development. For example, the fidelity findings indicate that there is a need for more thorough, accessible, and effective training of CHWs when it comes to both the intervention content and its delivery. Inaccurate reporting had a strong impact on data quality, demonstrating the need for enhanced training. This issue is especially pertinent when it comes to any expected research contributions, such as recruitment of eligible participants, carrying out the consent process with caregivers, and collecting basic questionnaire data or any other data CHWs are responsible for collecting. In particular, these concerns will need to be resolved if a definitive trial is carried out to ensure quality of outcome data, and that ethical standards are adhered to in obtaining caregivers’ informed and voluntary consent for participation. In addition to more comprehensive training, the findings suggest a need to develop specific strategies to monitor delivery and ensure the fidelity of the intervention in order to accurately evaluate the effectiveness of the intervention in the future [45]. One approach is to allow for greater flexibility in future evaluations [10], with the aim to use process evaluation methodology to examine how modifications influence delivery and outcomes.

This study raises questions about how to proceed based on findings from formative research, as no quantitative threshold or pre-specified progression criteria were set to determine whether to proceed to an effectiveness trial of the intervention. This is an often overlooked topic, and there are calls for increased transparency and guidance on this aspect of formative studies [11]. The qualitative methods employed, in combination with the assessment of multiple process evaluation concepts and a theoretical framework to guide this investigation, enabled multifaceted learning and a nuanced picture of the complexities of intervention delivery. While there were promising findings relating to acceptability and appropriateness, there were several concerning features in the data relating to feasibility, fidelity, and context that fundamentally speak against this particular delivery approach. Moreover, the findings support the optimisation of the intervention in the future, demonstrating that the chosen data collection and analysis methods were appropriate for fulfilling the aims of this study.

As SK was responsible for carrying out the entire study, it is important to consider the role of observer bias, which is a typical limitation of intervention and implementation research [46, 47]. Such observer or research bias is likely to result in the exaggeration of positive aspects at the cost of more critical observations. Moreover, as the data collection took place at the PHC facility, participants may have felt uncomfortable expressing critical views despite efforts to ensure privacy. This is particularly important to consider given that SK (a White European woman) was an obvious cultural outsider in the context of the research, making it potentially very challenging to build trust and rapport in a meaningful way [48, 49]. Numerous site visits offered ample opportunities for informal discussions and observation of the functioning of the WBOT programme more broadly, and the sharing of critical opinions in interviews and FGDs also suggests that participants were not limiting their responses solely to positive views. Nevertheless, the results described here can be characterised as a research team with a predominantly foreign pose, writing for a predominantly foreign academic gaze [50], thus lacking the full nuances that a truly local perspective could offer.

A specific limitation of this study was the weak reliability and quality of data collected by the CHWs. As this shortcoming was identified through other data sources, it can be interpreted as triangulation providing a level of protection from falsified data. In addition to being somewhat challenged by the intrinsic limitations detailed above, external factors such as the strikes also hindered the optimal delivery of the *Amagugu Asakhula* intervention. Nonetheless, this study provides many important insights to support future research and implementation.

## Conclusions

This feasibility study contributes to the optimisation and further testing of the *Amagugu Asakhula* intervention and can inform the development and implementation of other nurturing care interventions. Delivering the *Amagugu Asakhula* intervention through CHWs linked to a PHC facility was found to be acceptable to caregivers of preschool children. However, this delivery mode was not found to be feasible for further implementation and testing due to organisational constraints related to the WBOT system in South Africa. Delivery through CHWs who are linked to a community-based organisation is recommended while recognising challenges linked to scalability and sustainability. The effects of *Amagugu Asakhula* on child health and developmental outcomes should be evaluated through definitive trials accompanied by process evaluation.

## Supplementary Information

**Additional file 1:.** Data collection instruments

## Data Availability

The datasets generated and/or analysed during the current study are not publicly available due to participants not being asked for permission for making the data publicly available. Anonymous excerpts from transcripts are available from the corresponding author on reasonable request.

## Abbreviations

CHW: Community health worker
FGD: Focus group discussion
LMIC: Low- and middle-income countries
PHC: Primary health care
WBOT: Ward-based outreach team
