# Supplementary material for: Can public sector community health workers deliver a nurturing care intervention in South Africa? The Amagugu Asakhula feasibility study
Source: Pilot Feasibility Stud. 2021 Feb 27;7:60. doi: 10.1186/s40814-021-00802-6 (PMC7912559; doi:10.1186/s40814-021-00802-6)
Supplement: Supplementary file 1 — Additional file 1:. Data collection instruments [file 40814_2021_802_MOESM1_ESM.docx]

**Amagugu Asakhula caregiver questionnaire**

| Today’s date: | Caregiver ID: |
| --- | --- |
| Preschool child’s ID: | Caregiver’s date of birth: |
| Preschool child’s date of birth: | Preschool child gender: |

1. What relationship are you to the preschool child in this study? (tick ONE)

| 🞆 | Mother | 🞆 | Grandmother |
| --- | --- | --- | --- |
| 🞆 | Aunt | 🞆 | Other (please state): |

2. What is your current marital status? (tick ONE)

| 🞆 | Married | 🞆 | Living together | 🞆 | Divorced |
| --- | --- | --- | --- | --- | --- |
| 🞆 | Separated | 🞆 | Widowed | 🞆 | Never married |

3. What is your highest level of education? (tick ONE)

| 🞆 | Grade 6 / Standard 4 and below | 🞆 | Grade 7-9 / Standard 5-7 |
| --- | --- | --- | --- |
| 🞆 | Grade 10-11 / Standard 8-9 | 🞆 | Grade 12 / Standard 10 / Matric |
| 🞆 | Tertiary diploma / Certificate | 🞆 | University degree |

4. What is your home language? (tick ONE)

| 🞆 | English | 🞆 | isiXhosa | 🞆 | Zulu | 🞆 | Tsonga/Shangaan |
| --- | --- | --- | --- | --- | --- | --- | --- |
| 🞆 | Afrikaans | 🞆 | Sotho | 🞆 | Pedi | 🞆 | Tswana |
| 🞆 | Other (please state): | |  | | | | |

5. Please use the table below to tell us about the other children in your household:

| Other children | Child’s date of birth | Boy or girl? |
| --- | --- | --- |
| 1 |  | 🞆 Boy 🞆 Girl |
| 2 |  | 🞆 Boy 🞆 Girl |
| 3 |  | 🞆 Boy 🞆 Girl |
| 4 |  | 🞆 Boy 🞆 Girl |
| 5 |  | 🞆 Boy 🞆 Girl |
| 6 |  | 🞆 Boy 🞆 Girl |

6. What time does your child usually go to sleep at night, and wake up in the morning?

|  |  | School nights (Sun-Thurs) | Weekend (Fri & Sat) |
| --- | --- | --- | --- |
| Time to sleep at night |  |  |  |
|  |  | School mornings (Mon-Fri) | Weekend (Sat & Sun) |
| Time to wake up in the morning |  |  |  |

7. How often is there a bedtime routine for this child (e.g., bath time, saying goodnight, storytelling, etc)

| 🞆 | Never | 🞆 | Less than once a week | 🞆 | Once a week |
| --- | --- | --- | --- | --- | --- |
| 🞆 | Most days | 🞆 | Every day | 🞆 | Don’t know |

8. During the past week, on how many days did you or other household members read to this child?

| 0 | 1 | 2 | 3 | 4 | 5 | 6 | 7 |
| --- | --- | --- | --- | --- | --- | --- | --- |

9. During the past week, on how many days did your child have sweets and/or chips?

| 0 | 1 | 2 | 3 | 4 | 5 | 6 | 7 |
| --- | --- | --- | --- | --- | --- | --- | --- |

10. During the past week, on how many days did your child have fizzy drinks, juice and/or cooldrinks?

| 0 | 1 | 2 | 3 | 4 | 5 | 6 | 7 |
| --- | --- | --- | --- | --- | --- | --- | --- |

11. During the past week, how many fruits and vegetables did your child usually have every day?

| Fruits: |  | Vegetables: |  |
| --- | --- | --- | --- |

12. How much time does your child spend on screens in a typical 24-hour day?

This includes any electronic screen device such as a smart phone, tablet, video game, or watch television or movies, videos on the internet while they were sitting or lying down.

| Hours: |  | Minutes: |  |
| --- | --- | --- | --- |

Observation

(Done by CHW / fieldworker, with caregiver’s assistance)

Briefly describe the sleeping situation (e.g. All 4 family members sleep in 1 bed in the bedroom OR The caregiver and her husband sleep in the bedroom, and the 3 children sleep in the lounge on 2 beds).

|  |
| --- |

Tick in the boxes next to the toys or equipment you observe in caregiver’s home, and write down the amount/number that you observe (e.g. 2 TVs).

| Children’s books |  | Puzzles |  |
| --- | --- | --- | --- |
| Colouring books/pages |  | Crayons/koki pens/pencil crayons |  |
| Lego/Duplo/other building blocks |  | Board/card games |  |
| Other toys/games for quiet play: |  | Other toys/games for quiet play: |  |
| TV |  | Video/DVD player |  |
| Digital tablet, e.g. iPad |  | Smart phone |  |
| Video games, e.g. Playstation, X-Box |  | Satellite dish (for TV) |  |
| Computer |  | Internet access |  |

Thank you for completing this questionnaire!

| Amagugu Asakhula CHW activity log | | | | | | |
| --- | --- | --- | --- | --- | --- | --- |
| CHW’s name | Caregiver’s name | Session number | Date | Time of session | Length of session | Comments (e.g. who was present for the session) |
|  |  |  |  |  |  |  |
|  |  |  |  |  |  |  |
|  |  |  |  |  |  |  |
|  |  |  |  |  |  |  |
|  |  |  |  |  |  |  |
|  |  |  |  |  |  |  |

Focus group guide

Purpose of the focus group discussion:

The purpose of this focus group discussion is to hear what those who were involved in the Amagugu Asakhula intervention think about intervention – whether it was feasible and acceptable, and why. This includes the community health workers who delivered the intervention, as well as the caregivers were beneficiaries of the intervention.

Date: ______________________

Starting Time: _________ Ending Time: ___________

Please fill out and attach the participant register.

Name of Facilitator: ______________________________________________________

Name of Note Taker: _____________________________________________________

Name of Observer: _______________________________________________________

| - DIRECTIONS: The qualitative focus group begins with open-ended questions that point to starting a dialogue. The interviewer should encourage the study participants to do most of the talking but should use the questions listed here as a guide. After you ask each question, wait for the study participants to respond and go on to the next question when you are satisfied with the answer. If it seems as though the study participants did not understand a question, then repeat it or ask it in another way. If the study participants go on talking without much prompting, then let them guide the conversation. Bold indicates major questions and probes are in parentheses. Mentally check off these questions as they are asked so you do not repeat a question if it has been discussed previously. - Remember to audio record each focus group. Use a backup recorder to prevent technological problems.   Note to the Facilitator:  Introduce yourself at the beginning of the session, explain who you work with, why you are here, and introduce everyone on the team who is with you observing, taking notes, or helping in anyway.  Introduction: Hello, my name is ________________________________. I am working with researchers at the Developmental Pathways to Health Research Unit (DPHRU) on the Amagugu Asakhula intervention. We are interested in hearing what you think about Amagugu Asakhula. This should not take more than about one hour. Do not worry. There are NO right or wrong answers. Your ideas and answers to our questions are very important to us. You are free to join this group discussion, and free to answer or not to answer the questions we are going to ask. You should feel very free to express whatever you are thinking. Your responses are confidential and anonymous. Please feel free to ask for clarification if you do not understand a question. Do you agree to join this group?  (Introduce the others on the team) Do we have your permission to continue? |
| --- |

| Note to Note-Taker: Try to capture the major ideas and something about the majority of participants agreeing or not agreeing. Always note the specific question that the facilitator and participants are referring to. If the facilitator asks a question that is not on the guide, note the question as it is asked and try to capture the answers. If you need more space, use the extra paper and note the name of the group and the corresponding number of the question.  Note to Observer: You can take notes about the answers also, but focus on the dynamics of the group and how people are reacting to the questions and to the discussion. If you can, make a note about who are the most active participants so that we can follow-up with them. |
| --- |

Guide questions:

- Overall, what did you think of Amagugu Asakhula?
- How did you feel being part of this programme?
- What did you feel you learnt about the concepts that were covered in the programme, e.g. cognitive development, physical activity, caregiver’s role in promoting their child’s health and development.
- Did these concepts make sense to you?
- Did you feel these concepts were relevant for caregivers of preschool children in your community?
- How did you think the programme worked in terms of all the practical things, such as:
  - Scheduling a regular session, once per week
  - Having home-based sessions
  - The length of sessions
  - The number of sessions
  - Anything else?
- Which the activities and materials did you like, or think went well? Prompt:
  - Tree picture
  - Bean growing kit
  - Family map and materials
  - Flashcards
  - Screen time, sleep and food diaries
  - Blackboard for star chart
- Do you think that any of the activities did not go well / materials did not work?
- Were there any challenges that you experienced with the programme?
- Only community health workers: How did you feel about the training you received? Was there any additional support that you required?

Key informant interview guide

Purpose of the interviews:

The purpose of these interviews is to hear what key informants have to say about the feasibility and acceptability of the intervention, as well as the appropriateness and potential adoption, coverage and sustainability of integrating the intervention into community health workers’ scope of work.

Date: ______________________

Starting Time: _________ Ending Time: ___________

Name of Interviewer:

______________________________________________________

Name of Interpreter (if present):

_____________________________________________

| - DIRECTIONS: The qualitative interview consists of open-ended questions that allow caregivers to describe their views and experiences in their own words. The interviewer should encourage the study participants to do most of the talking but should use the questions listed here as a guide. After you ask each question, wait for the study participants to respond and go on to the next question when you are satisfied with the answer. If it seems as though the study participants did not understand a question, then repeat it or ask it in another way. If the study participants go on talking without much prompting, then let them guide the conversation. Mentally check off these questions as they are asked so you do not repeat a question if it has been discussed previously. - Remember to audio record each interview. Use a backup recorder to prevent technological problems.   Note to the Interviewer:  Introduce yourself at the beginning of the session, explain who you work with, why you are here, and introduce everyone on the team who is with you observing, interpreting, taking notes, or helping in anyway.  Introduction: Hello, my name is ________________________________. I am working with researchers at the Developmental Pathways to Health Research Unit (DPHRU) on the Amagugu Asakhula intervention. We are interested in hearing what you think about Amagugu Asakhula. This should not take more than about one hour. Do not worry. There are NO right or wrong answers. Your ideas and answers to our questions are very important to us. You are free to answer or not to answer the questions we are going to ask. You should feel very free to express whatever you are thinking. Your responses are confidential and anonymous. Please feel free to ask for clarification if you do not understand a question. Do you agree to participate in this interview? Do we have your permission to continue? |
| --- |

Guide questions:

- What are your impressions of the feasibility of the Amagugu Asakhula intervention?
  - How do you think it works with community health workers?
  - How do you think they were able to integrate it into their work with families?
- What are your impressions of the acceptability of the intervention?
  - How acceptable do you think it was to community health workers?
  - How acceptable do you think it was to families?
- How scalable do you think the intervention is?
- What do you think about the potential adoption of this intervention into usual care of community health workers?
- What do you think about the reach or coverage of this intervention, if it’s delivered by community health workers?
  - How do you think this would differ in different communities?
- Do you think the intervention could be taken up by government, or do you think this should be driven by the NGO sector?
- Do you have any other feedback or suggestions about the intervention?
